# Cadherin-12 contributes to tumorigenicity in colorectal cancer by promoting migration, invasion, adhersion and angiogenesis

**DOI:** 10.1186/1479-5876-11-288

**Published:** 2013-11-15

**Authors:** Jingkun Zhao, Pu Li, Hao Feng, Puxiongzhi Wang, Yaping Zong, Junjun Ma, Zhuo Zhang, Xuehua Chen, Minhua Zheng, Zhenggang Zhu, Aiguo Lu

**Affiliations:** 1 Shanghai Minimally Invasive Surgery Center, Shanghai Key Laboratory of Gastric Neoplasms, Department of Surgery, Shanghai Institute of Digestive Surgery, Ruijin Hospital, School of Medicine, Shanghai Jiao Tong University, 197 Rui Jin Er Rd, Shanghai 200025, People’s Republic of China

**Keywords:** Cadherin12, Colorectal cancer, Cell proliferation, Cell migration, Cell invasion, Cell adhesion, Angiogenesis

## Abstract

**Background:**

Cadherin 12 (CDH12), which encodes a type II classical cadherin from the cadherin superfamily, may mediate calcium-dependent cell adhesion. It has been demonstrated that CDH12 could play an important role in the invasion and metastasis of salivary adenoid cystic carcinoma. We decided to investigate the relationship between CDH12 expression level and clinicopathologic variables in colorectal carcinoma (CRC) patients and to explore the functions of CDH12 in tumorigenesis in CRC.

**Methods:**

The expression levels of CDH12 in colorectal carcinoma tissues were detected by immunohistochemistry. Real-time PCR and Western Blot were used to screen CDH12 high-expression cell lines. CCK-8 assay was used to detect the proliferation ability of CRC cells being transfected by shRNAs against CDH12. The wound assay and transwell assay were performed to test migration and invasion ability. The importance of CDH12 in cell-cell junctions was detected by cell adhesion assay and cell aggregation assay. Endothelial tube formation assay was used to test the influence of CDH12 on angiogenesis.

**Results:**

Statistical analysis of clinical cases revealed that the positive rate of CDH12 was higher in the CRC tumor tissues compared with the adjacent non-tumor tissues. The expression levels of CDH12 in CRC patients are significantly correlated with invasion depth. Consistently, the ability of proliferation, migration and invasion were suppressed when CDH12 was decreased in CRC cells transfected with shRNAs. Cell adhesion assay and cell aggregation assay presented that tumor cells tend to disperse with the lack of CDH12. Endothelial tube formation assay showed that down-regulation of CDH12 could obviously inhibit the process of angiogenesis, implying that CDH12 may play an important role in tumor metastasis

**Conclusion:**

Our results showed that CDH12 promotes proliferation, migration, invasion, adhesion and angiogenesis, suggesting that CDH12 may be an oncogene in colorectal cancer. CDH12 is expected to become a new diagnostic and prognostic marker and a novel target of the treatment of colorectal cancer.

## Background

Cadherin adhesion molecule is one of the ubiquitous types of cell-cell interactions required for the maintenance of complex tissue morphology. Traditionally, cadherins are membrane-spanning Ca^2+^-dependent homophilic adhesion receptors including E-cadherin, N–cadherin, P-cadherin, VE-cadherin [1]. Classical cadherin molecules, especially E-cadherin and N-cadherin are involved intimately in the pathogenesis of various cancers such as breast cancer, esophageal cancer, prostate cancer, gastric cancer *etc.*[2-5]. The loss of E-cadherin and the up-regulation of N-cadherin influence the behaviors of many types of cancers which process is also known as cadherin switch [6]. Considering the important function of cadherin in cell-cell contacts, they are usually postulated to play a vital role in cancer metastasis.

Colorectal cancer is one of the most common cancer types worldwide and its incidence increases with the change of life style and dietary structure [7]. Colorectal cancer has the propensity to colonize the liver and leads to a poor prognosis in the patient. However, metastasis is a complex process and has not been fully understood. We hypothesized that the destruction of cadherin adhesions acts as a promoter to facilitate the metastasis of colorectal cancer. In this article, we identified CDH12 as such an important molecule. CDH12 is also known as brain cadherin, because it is first identified in the brain and it can promote the differentiation of nervous system in mammary animal. In addition, CDH12 is associated with the pathogenesis of some neuropsychiatric disorders [8,9]. The comprehensive influence of classical cadherins on cancer such as E-cadherin and N-cadherin has been deeply clarified. However, the impact of CDH12 on cancer didn’t attract more attention until recent years. Gemma Armengol *et al*. verified that CDH12 plays a significant role in the progression of non-small-cell lung cancer, and patients without CDH12 mutations have a longer survival than those with CDH12 mutation. This indicates that CDH12 acts as a tumor suppressor gene in the non-small-cell lung cancer [10]. In addition, CDH12 is able to promote the migration and invasion ability of salivary adenoid cystic carcinoma [11]. However, the function of CDH12 in colorectal cancer is unclear. In our research, we demonstrated the down-regulation of proliferation, invasion and migration ability in colorectal cancer cell lines by silencing CDH12 expression. Furthermore, we showed down-regulation of CDH12 can obviously inhibit the process of angiogenesis, implying that CDH12 may play an important role in tumor metastasis.

## Methods

### Cell culture

Seven human CRC cell lines were purchased from American Type Culture Collection (Manassas, VA, United States). All the cell lines are preserved by Shanghai Digestive Surgery Institute. SW1116, SW480 and SW620 are cultured in Leibovitz’s L-15 medium supplemented with 10% fetal calf serum, and 2 mM L -glutamine. HCT116, HT29, Caco2, LoVo and NCM460 were maintained in RPMI-1640 medium with the same components. CRC cell lines were cultured in 37°C, 5% CO_2_ incubator. Total RNA and protein were extracted after being incubated to 70-80% confluence.

### Tissue microarray and immunohistological analysis

Tumor tissue and adjacent normal tissue of 56 patients with specific diagnosis of CRC and laparoscopic surgery in Minimally Invasive Surgery Centre, Ruijin Hospital,Shanghai Jiaotong University were collected from 2009 to 2011. The collection of specimens was authorized by Ethics Committee of Ruijin Hospital. None of the patients received preoperative treatment such as radiation or chemotherapy. The patients include 33 males, 23 females and the median age is 65.Staging of CRC tumor tissue was performed in accordance with the TNM classification provided by World Health Organization [12]. All the specimens fixed by 10% formaldehyde and embedded with paraffin were made into microarray. The staining of tissue microarray was conducted according to the manufacturer’s protocol. After being dewaxed and hydrated, the microarray was antigen-retrieved by microwaving in citrate buffer (10 mM citric acid, pH 6.0), blocked in 5% animal serum, and stained for 2 h at 37°C by using primary antibody. Next ,the microarray was stained for 10 min at 37°C by using secondary biotinylated anti-rabbit antibodies at a 1:100 dilution followed by streptavidin-HRP. Specimens were developed by DAB and the nucleus was counterstained by Hematoxylin. The sections were photographed under a microscope and analyzed by two pathologists without knowing the pathological materials. Staining results were evaluated following the standard provided by Shimazui T et al. [13,14]. When the CDH12 were uniformly expressed, heterogeneously expressed or none-expressed in the tissue, the sections were evaluated as uniformly CDH12 positive (+), heterogeneous (+/−), or uniformly negative (−), respectively.

### Quantitative reverse transcription polymerase chain reaction

Total RNA was extracted with TRIzol (Invitrogen, Carlsbad, CA, United States) and the concentration was measured by spectrophotometer. Reverse transcription was performed to synthesize cDNA using reverse transcription kit(Invitrogen, CA, United States). The reaction was performed in an Applied Biosystems 7500 System with the mixture of cDNA, primers and Power SYBR Green PCR Master Mix (2×, Applied Biosystems, Warrington, United Kingdom).CDH12 PCR sense primer: 5′-AGGAGGTGGGGAGGAAGATA-3′, antisense primer: 5′-CATATGTGGCCAGTGAATCG-3′. To quantify the expression level of CDH12, we use GAPDH as a control. GAPDH PCR sense primer: 5′-CCACGGAGCCGAAAACTAAAG-3, antisense primer: 5′-GTAGCCCAGGATGCCCTTGA-3′.

### Western blot analysis

Protein lysates were prepared from collected cells with RIPA and the supernatant was collected after being reacted for 30 min on the ice, centrifuged for 15 min at 13000 rpm. BCA (bicinchonininc acid) was used to determine protein concentration. An equal amount of protein (50 μg) from each condition was subjected to 12.5% SDS-PAGE. Western blotting was carried out using the primary antibodies: rabbit polyclonal anti-CDH12 antibody (Abcam), 1:100; mouse polyclonal anti–GAPDH antibody,1:5000 for overnight at 4°C, followed by incubation with fluorescence-labeled secondary antibody for 2 h at room temperature. LI-COR Odyssey Infrared fluorescence scanner was used to capture the images.

### RNAi

3×10^5^ cells were seeded into six-well plate each chamber. Until 70% confluence, GFP-labeled shRNA targeting CDH12 was transfected into cells mediated by Lipofectamine^TM^ 2000 according to the instruction. Cell sections transfected with GFP-labeled shRNA targeting nonhomologous gene to CDH12 and Lipofectamine^TM^ 2000 only were performed as control. After 12 hours transfection, cells were observed under fluorescence microscope.

### ShRNA oligonucleotides screen

The following shRNA oligonucleotides were used to interfere CDH-12 expression and the most effective oligonucleotide was screened with determining the mRNA level using quantitative PCR:shCDH12-1: 5′-CACCGCTGGGCAACAATTCTCCTTT

TCAAGAGAAGGAGAATTGTTGC CCAGCTTTTTG-3′(sense sequence),

shCDH12#2:5′- CACCGCGCAGTATAATTTCTCCATAC TCGAGTATGGAGA

AATTATACTGCGCTTTTTG-3′(sense sequence),

shCDH12#3:CACCCGGTCACATTTCCAACGTGTTCTCGAGAACACGTTGGAAATGTGACCGTTTTTG-3′(sense sequence),

Negative control: 5′-CACCGT TCTCCGAACGTG TCACGTCAAGAG ATTACGTG ACACGTTCGGAGAATTTTTTG-3′(sense sequence).

### Cell viability assay

Cells were seeded in 96-well plate and cultured in 37°C, 5% CO_2_ incubator. Three groups (shCDH12 group, shNC group and Mock group) were designed with 6 copies each group. The viability of cells was determined at 0 h, 24 h, 48 h, 72 h, 96 h and 120 h. At the test point, 10 μL CCK-8 was added into each well and the plate was incubated at 37°C for 2 h followed OD detection using spectrophotometer.

### Cell invasion and migration assay

Transwell chamber was used (8 mm, 24-well format; Corning, Lowell, MA, USA) to perform cell invasion and migration assay. In migration assay, 200 μL Leibovitz’s L-15 serum-free medium containing 3×10^5^ cells was added into the upper chamber and 600 μL Leibovitz’s L-15 with 10% serum was added into the lower chamber. The chamber was cultured in 37°C, 5% CO_2_ condition for 24 h. In invasion assay, the insert membranes were coated with diluted Matrigel (BD Biosciences) and performed in the same way as migration assay. Finally, cells on the top side of the inserts were removed with cotton swab. Chambers were fixed with methanol and Cells under the inserts were stained with 1% crystal violet for 30 min before rinsing with phosphate buffered saline (PBS) for 20 min. The results are shown as means ± sd. Each experiment was done in triplicate. The wound healing assays was performed as previously described [15,16].

### Flow cytometric cycle analysis

CRC cells were collected after 24 h, 48 h respectively and rinsed by PBS for twice. Collected cells were fixed in 75% iced ethanol for detection. Fixed cells was recollected by washing in PBS for twice, centrifuging at 1800 rpm for 5 min. RNAase (3 μL) and Propidium iodide (PI, 50 μL) were added into each tube, incubated for 30 min at 37°C in the darkness. The Cell cycle analysis was performed using BD FACS Vantage System (Becton, Dickinson and Company, Franklin Lakes, NJ, United States) according to the manufacturer’s protocol.

### Cell aggregation assay

Cells were harvested with trypsin/EDTA and resupended at 1.5×10^5^ cells per ml in RPMI-1640 medium with FBS. 20 μL drops containing 5000 cells per drop were pipetted onto the inner surface of the lid of petri plate. The lid were then reversed and placed on the petri plate with the drop hanging on the lid so that the cells failed to adhere to the petri plate. 4 ml of PBS was placed in the petri plate to eliminate evaporation within the hanging drops and the results were observed under microscope.

### Cell adhesion assay

Human umbilical vein endothelial cells (HUVEC) were plated into 24-well plate and incubated at 37°C, 5% CO_2_ for 24 h to form monolayer. Tumor cells after being managed respectively were collected with trypsin/EDTA and resuspended by RPMI-1640 medium. 700 μL medium containing 20,000 cells were subsequently added into each well and incubated in the incubator for 6 h. Subsequently, medium in each well was abandoned and the wells were washed by PBS for three times. Results were observed under fluorescence microscope and five random views were chosen to quantify.

### Endothelial tube formation assay

Each well of prechilled 96-well plates was coated with a thin layer of the Matrigel (50 μL/well) which was incubated to polymerize at 37°C for 1 h. Human umbilical vein endothelial cells (HUVEC) were resuspended in the supernants collected from shRNA group, control groups. Add 300 μL of the supernants to each well containing 4×10^4^ HUVEC cells and incubated at 37°C, 5% CO^2^ for 24 h. Five random views were choosed to evaluate tube formation ability by counting the tubular number, the tubular length and tubular intersecting nods in using Image Pro Plus software (Media Cybernetics Inc., Bethesda, MD, USA) according to Mirshahi’s method [17]. Each experiment was performed at least three times.

### Statistical analysis

All the data were analyzed by SAS 8.0 statistical software. The difference of CDH12 expression between tumor tissue and adjacent normal tissue was examined by Cochran- Maantel-Terpstra test. Chi-square test and Fisher exact probability method was used to analyze the relationship between CDH12 expression level and clinical features. Survival probabilities were calculated using Kaplan–Meier method, differences between two groups were determined using the log-rank test. Multiple comparisons were performed by one-way analysis of variance. *P*<0.05 was considered statistically significant.

## Results

### The expression levels of CDH12 in CRC patients are significantly correlated with invasion depth.

**Figure 1:**
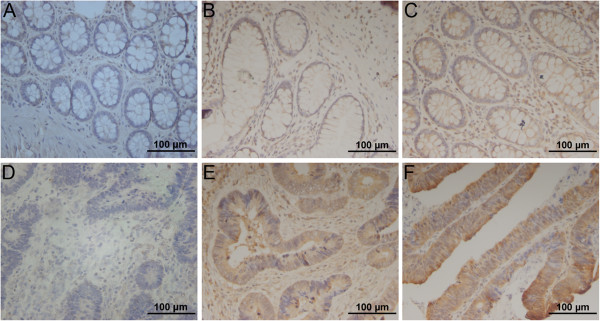
**Immunohistochemical results of CDH12 expression in surgical specimens of tumor tissues or adjacent normal tissue from patients with colon cancer. A-C**: Uniformly negative (−), heterogeneous (+/−), and uniformly positive (+) staining in adjacent normal mucosa(200×); **D-F**: Uniformly negative (−), heterogeneous (+/−), and uniformly positive (+) staining in CRC tumor tissues (200×).

Immunohistochemistry tissue microarray staining was performed to detect the expression of CDH12 in the CRC tissue, and the representative results were shown in Figure 1. 41 cases out of the 56 tumor samples exhibited a substantial increase in overall CDH12 expression, 10 cases expressed heterogeneously and 5 cases expressed uniformly negative. Obvious difference between tumor tissues and adjacent normal tissues was verified by statistical analysis, the positive rate is significantly higher in the tumor tissues compared to the adjacent normal tissues (Table 1). The expression level of CDH12 is significantly correlated with invasion depth (*P*=0.024). However, there are no significant relationships between the CDH12 expression and other clinicopathologic features such as gender, age, tumor size, or lymph node metastasis (*P*>0.05) (Table 2). In addition, to further elucidate the clinico-pathologic significance of CDH12 overexpression in CRC with prognosis, we correlate CDH12 expression in CRC tissues with the survival time 44 out of 50 patients that were successfully followed up. The Kaplan-Meier survival analysis showed the survival rate of the CDH12 positive patients group was significantly lower than the CDH12 negative patients group (*P*<0.05, Additional file 1: Figure S1).

**Table 1:** The expression of CDH12 in tumor tissues and adjacent normal tissues

**Classification**	**n**	**CDH12(−)**	**CDH12(+/−)**	**CDH12(+)**	***P *** **value**
Tumor tissue	56	5		41	<0.001
Normal tissue	56	23	24	9	

**Table 2:** Relationship between CDH12 expression level and clinicopathologic variables in 56 CRC patients

**Classification**	**CDH-12(−)**	**CDH-12(+/−)**	**CDH-12(+)**	***P *** **value**
Gender				
Male	3	7	23	0.725
Female	2	3	18	
Age (year)				
≤65	2	4	19	0.915
>65	3	6	22	
Tumor location				
right hemicolon	0	1	11	0.283
left hemicolon	1	1	1	
sigmoid colon	2	4	8	
rectum	2	4	21	
Tumor size (cm)				
≤4×3	3	7	23	0.725
>4×3	2	3	18	
TNM stage				
invasion depth				
T_1_	2	0	1	0.024
T_2_	1	1	4	
T_3_	2	8	34	
T_4_	0	1	2	
lymph node				
N_0_	1	5	18	0.237
N_1_	4	2	16	
N_2_	0	3	7	
organ metastasis				
M_0_	4	10	38	0.365
M_1_	1	0	3	

### Expression of CDH12 in CRC cell lines and the interfering effect of shRNA

**Figure 2:**
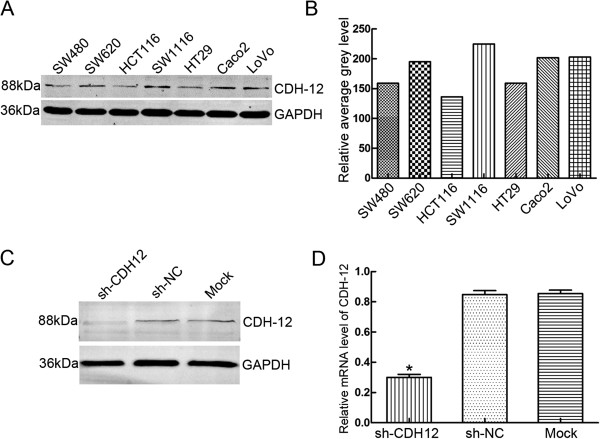
**Expression of CDH12 in CRC cell lines and the interfering effect of shRNA. A**: CDH12 expression in CRC cell lines detected by western blot; **B**: Relative average grey level of western blot bands; **C**: Interfering effects of shCDH12 in SW1116 were detected using western blot; **D**: mRNA expression levels of CHD12 in SW1116 cell lines transfected with shRNAs (*P<0.05).

Western blot was used to detect the CDH12 expression in CRC cell lines, including the non-cancer cell line NCM460. CDH12 was expressed in all these cell lines, and especially high in SW1116 and SW620, but low in HT29 and HCT116 (Figure 2B&C, Additional file 2: Figure S2B&C). In order to explore the functions of CDH12 in CRC cell lines, we chose the CHD12 high-expression cell lines SW1116 and SW620. The three shRNAs targeting CDH12 or a scramble control shRNA was transfected into SW1116 or SW620 and the expressions of CDH12 were detected (Additional file 2: Figure S2A). The results showed that the expression of CDH12 was significantly decreased in shRNA-CDH12 transfection cells compared with the control groups (Figure 2C, 2D).

### Down-regulation of CDH12 can inhibit CRC cells proliferation and block cell cycle progression

**Figure 3:**
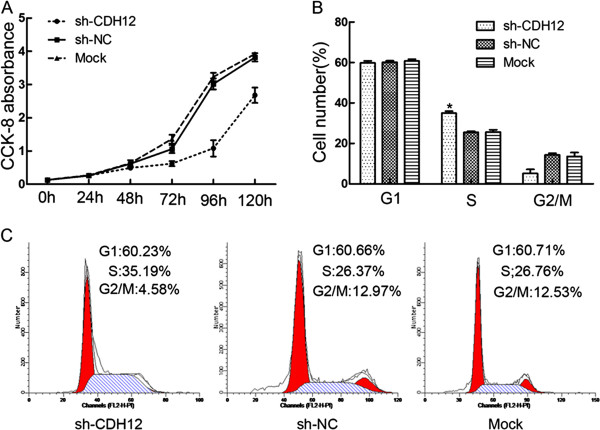
**Effect of CDH12 on cell proliferation and cell cycle in CRC cell lines. A**. Cell numbers at 0, 24, 48, 72,96 and 120 h was measured by CCK-8 assays. **B**: Proportion of cells in various phases of the cell cycle. The results are means of three independent experiments ± S.D (*:*P*<0.05). **C**: Representative histograms depicting cell cycle profiles of cells transiently transfected with shCDH12 or controls.

We next determined whether CDH12 could affect the cell proliferation and cell cycle. The proliferation ability of SW1116 was examined at six time points (0 h, 24 h, 48 h, 72 h, 96 h and 120 h) after being transfected with shRNAs. The results was shown as means ± SD (Table 3) and the proliferation curves (Figure 3A). Cell proliferation ability in shCDH12 group was decreased significantly compared with the control groups (*P*<0.01). Consistently, ectopic expression of CDH12 in HT29 and HCT116 promotes the proliferation of the CRC cells (Additional file 3: Figure S3A&D, *P*<0.01).

**Table 3:** The absorbance in the proliferation assay

**Group**	**24 h**	**48 h**	**72 h**	**96 h**	**120 h**
shCDH12	0.28±0.038	0.49±0.060	0.62±0.078	1.08±0.245	2.68±0.531
shNC	0.33±0.023	0.62±0.108	1.06±0.121	3.02±0.168	3.82±0.125
Mock	0.38±0.105	0.63±0.095	1.36±0.131	3.23±0.125	3.92±0.043

To further detect the influence of CDH12 on cell cycle, we performed flow cytometric cell cycle analysis to examine which phase of cell cycle was influenced by CDH12 in SW1116. Flow cytometric analysis demonstrated that proportions of cells in shCDH12 group at S phase is more than that in shNC group and Mock group (*P<0.05, Figure 3B&C). These data indicated that down-regulation of CDH12 could obviously block cells in S phase.

### Down-regulation of CDH12 inhibits wound healing ability, migration and invasion of CRC cells

**Figure 4:**
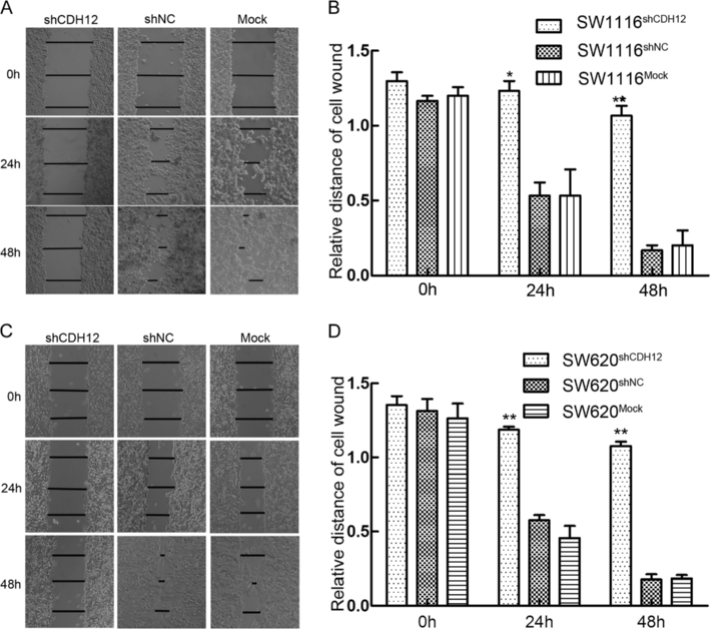
**Wound healing assay. A**: Representative photographs of scratch wounds in SW1116 (100×), **B**: Relative distances of cell wounds in shCDH12, shNC and Mock group (*:*P*<0.05), **C**: Representative photographs of scratch wounds in SW620 (100×); **B**: Relative distances of cell wounds in shCDH12, shNC and Mock group (**:*P*<0.01).

Wound healing assay was used to examine migration ability of SW1116 and SW620. Cells was plated in 12-well plate after being transfected and scratched by 20 μL sterile pipet tips. Results were showed in Figure 4A&C. Three lines were drawn in each group and relative length were calculated which is showed in Figure 4B&D. After 48 h incubation, the distance of the scratch woud in shCDH12 group is significantly larger compared with control groups. (The woud diatance: shCDH12 group: 1.07±0.11, shNC group: 0.17±0.06, Mock group: 0.20±0.17), The similar results were observed in theSW620 cell line (shCDH12 group: 1.08±0.05, shNC group: 0.18±0.06, Mock group: 0.18±0.04). Consistently, ectopic expression of CDH12 in HT29 and HCT116 promotes wound healing ability of CRC cells. (HT29, CDH12 group: 0.18±0.05, Vector group: 0.99±0.13; HCT116, CDH12 group: 0.20±0.06, Vector group: 0.83±0.07,) (Additional file 3: Figure S3 B&C and E&F).

**Figure 5:**
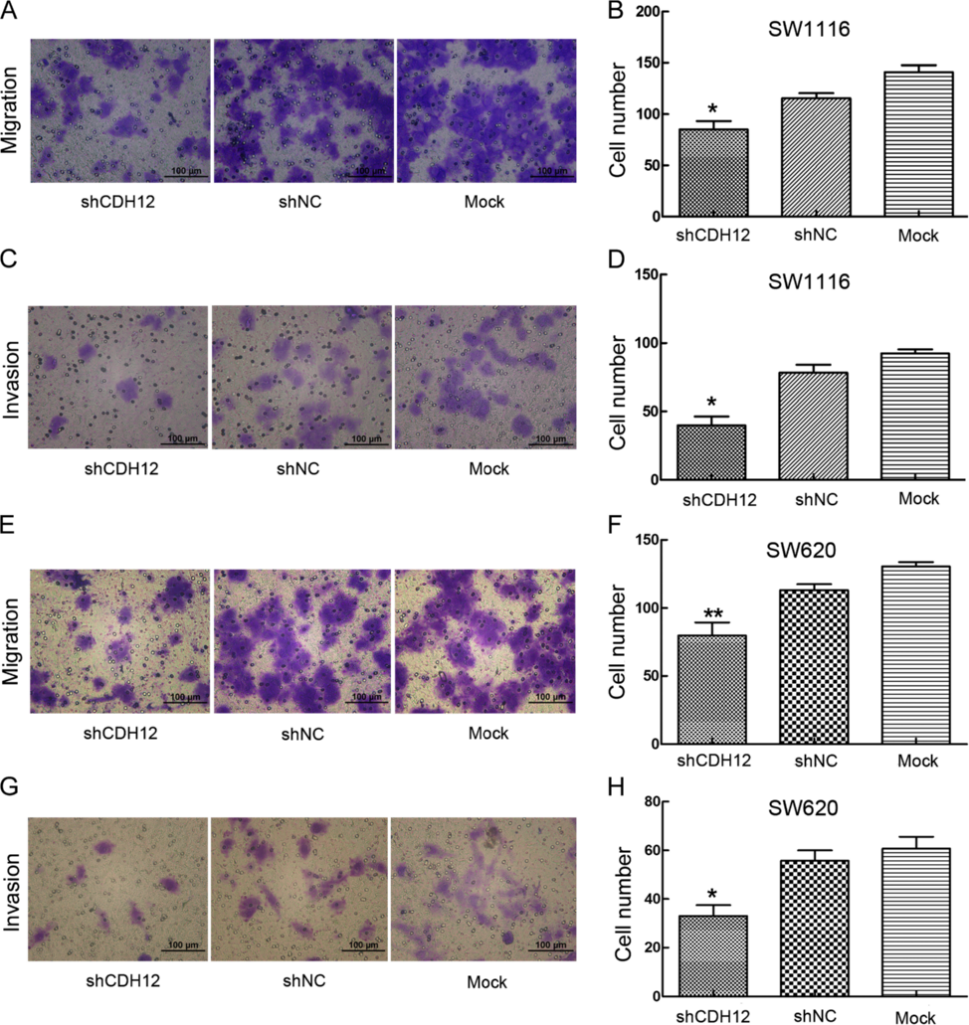
**Transwell cell migration and invasion assay. A**: Representative photographs of migratory SW1116 cells on the membranes. **B**: Cell numbers were counted at random five views, the average number of migratory cells were shown as histogram. (*:*P*<0.05). **C**: Representative photographs of invasive SW1116 cells on the membranes. **D**: Cell numbers were counted at random five views, the average number of invasive cells were shown as histogram. (*:*P*<0.05), **E**: Representative photographs of migratory SW620 cells on the membranes. **F**:Cell numbers were counted at random five views, the average number of migratory cells were shown as histogram. (**:*P*<0.01). **G**: Representative photographs of invasive SW620 cells on the membranes. **H**: Cell numbers were counted at random five views, the average number of invasive cells were shown as histogram. (**:*P*<0.01).

To further detect the influence of CDH12 on migration and invasion, we performed transwell migration and invasion assay. In transwell migration assay, the number of cells migrating through the chamber in shCDH12 was 85±18.42 which was lower than in shNC group (115.6±11.26) and mock group (141±15.17). The same result was also observed in the invasion assay: shCDH12 group: 37.80±11.28, shNC group: 78.40±13.05, Mock group: 93.60±8.91 (Figure 5A-D, *P<0.05). Similarly, the results are implemented in SW620 cell lines. (Migration assay: shCDH12 group: 78.4±10.38, shNC group: 120±8.7, Mock group: 135±9.8.sh; Invasion assay: shCDH12 group: 33±10.07, sh-NC group: 55.8±9.52, Mock group: 60.8±10.87). (**P<0.05, Figure 5E-H). Consistently, ectopic expression of CDH12 in HT29 and HCT116 promotes the migration and invasion of CRC cells (Additional file 4: Figure S4, *P<0.05, **P<0.01).

### CDH12 can promote cell-cell junction formation

**Figure 6:**
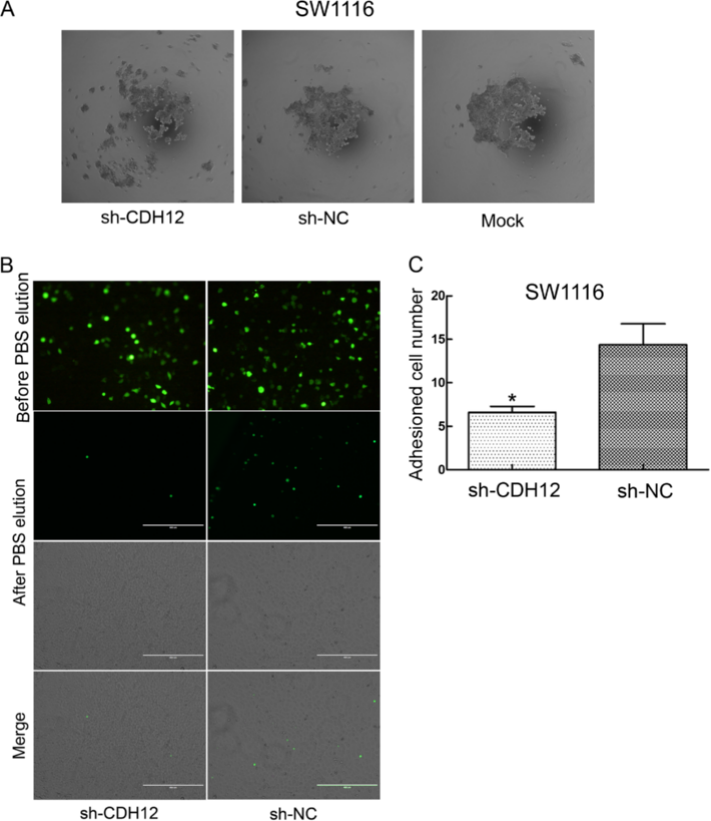
**Cell aggregation assay and cell adhesion assay. A**:The formation of cell colony was obviously inhibited in shCDH12 group compared with control groups. **B**: Cell numbers adhered to HUVEC monolayer in shCDH12 group is less than negative control group after PBS elution which demonstrated the function of CDH12 in tumor cell and endothelial cell junction. As the transfectant (shRNA and scrambled shRNA) is marked with GFP, so the cells adhered to the monolayer was showed as green dots. **C**: The numbers of the cells were counted in five random views and the statistical result is presented as histogram (*P<0.05).

As a member of the cadherin family, CDH12 is likely to influence the cell-cell junction. We firstly performed the cell aggregation assay to examine the role of CDH12 in cell-cell junction. The results demonstrated that suppression of CDH12 can inhibit the formation of cell colony because the cells in CDH12 knock-down groups distribute more dispersedly compared with control groups (Figure 6A). We performed adhesion assay subsequently to examine the adhesion ability of SW1116 to endothelial cells after CDH12 being decreased. After being washed with PBS, the number of SW1116 cells adhered to the HUVEC monolayer in the shCDH12 group was less than the shNC group (Figure 6B, 6C).These suggest that CDH12 is an essential adhesion molecule not only to tumor cells junctions but also to the adhesion between tumor cells and endothelial cells.

### CDH12 can promote endothelial tube formation

**Figure 7:**
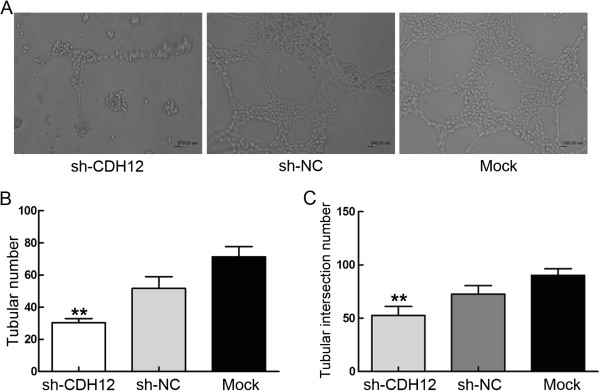
**Endothelial tube formation assay.** Effect of suppressing CDH12 expression on tubular formation *in vitro*. The supernatants from cells with or without CDH12 suppression were collected which was used to suspend HUVEC. After 24 h co-cultivation, tubular formation was evaluated. **A**: Poorly formed tubular structure was observed in shRNA group, compared with negative control group and mock group. **B**: The chart represent the counting of tubules numbers (**P<0.001); **C**: The intersecting nods (**P<0.001), The data represent the mean±SD of three independent experiments.

Human umbilical vein endothelial cells (HUVECs) (4×10^4^cells/well) were suspended in supernatants collected from shCDH12 group, shNC group and Mock group. After being incubated for 24 h, tubular numbers of each group were assessed under microscope. The supernatant from shCDH12 group presented strong inhibiting effect on endothelial tube formation compared with shNC group and Mock group whatever in tubular number and tubular intersecting nods (Figure 7).

## Discussion

CDH12 protein belongs to type II transmembrane protein containing 794 amino acids with a molecule weight of 88kDa and functions in the Ca^2+^-dependent cell-cell junction. CDH12 can promote the migration and invasion ability of salivary adenoid cystic carcinoma [11]. According to our findings, CDH12 may be an oncogene and promote the malignant progression of CRC cells.

Our findings showed that CDH12 expression is substantially upregulated in CRC tissues compared to adjacent normal tissue according to the histochemistry microarray analysis, indicating that CDH12 may also contribute to tumorigenicity in CRC. In addition, the expression of CDH12 is significantly associated with invasion depth. These data suggests that CDH12 may be associated with CRC invasion and metastasis. We selected the CDH12 high-expression cell lines SW1116 and SW620, and successfully inhibited CDH12 expression in them by shRNA. After CDH12 abrogation, the migration and invasion ability of SW1116 or SW620 was severely impaired.

These results verified the important role of CDH12 in CRC cells migration and invasion behavior and which may be explained that CDH12 is related to several intracellular metastasis-associated signal molecules that can promote tumor cell migration. This is consistent with the report that CDH12 is able to promote the migration and invasion ability of salivary adenoid cystic carcinoma [11].

We also performed cell aggregation assay and adhesion assay to further determine the importance of CDH12 in intercellular junction. Cell aggregation assay showed that the absence of CDH12 leads cells to distribute more dispersedly compared with control groups. Destruction of cell-cell junction is an initial reaction of tumor cell metastasis which manifests with repression of cell adhesion molecules. This process may be also known as epithelial-mesenchymal transition (EMT) [18]. Reduced intracellular adhesion may allow tumor cells to disseminate and spread throughout the extracellular matrix. Generally, tumor cells gaining the ability to leave the site and metastasize are accompanied with “cadherin switching” [6]. CDH12 may also promote tumor metastasis through this switching mechanism. Simultaneously, adhesion assay that cells numbers attached to the HUVEC monolayer in shCDH12 group were less than the control group, revealed the importance of CDH12 in the adhesion of tumor cells to endothelial cell. In the process of tumor metastasis, the arrest of circulating tumor cells to luminal walls of distant organ microvasculature is an essential part of this process [19]. Collectively, the result demonstrates that CDH12 may promote tumor cells to attach to the vascular enthelial cells during tumor metastasis.

Tumors require supplements of nutrients and oxygen and evacuation of metabolic wastes and carbon dioxide as normal tissues. The tumor-associated neovasculature, generated by the process of angiogenesis, not only supplies abundant nutrients for tumor cell but also provides conveniences for tumor cell dissemination [20]. Our results showed that down-regulation of CDH12 on SW1116 or SW620 cells effectively inhibited the formation of vessel. The numbers of tube, tubular length and tubular intersecting nods in shRNA group is less than the control. This suggests that CDH12 can promote the process of angiogenesis. Considering the importance of angiogenesis in tumor metastasis and the contribution of CDH12 in tumor cell migration, invasion and cell cycle, CDH12 maybe a potential target to supervise tumor cell malignant metastasis.

## Conclusion

Our results showed that CDH12 promotes proliferation, migration, invasion, adhesion and angiogenesis, suggesting that CDH12 may be an oncogene in colorectal cancer. CDH12 is expected to become a new diagnostic and prognostic marker and a novel target of the treatment of colorectal cancer. More efforts should be put to clarify the signal pathways underlying these biological phenomenon.

## Abbreviations

CDH12: Cadherin 12; CRC: Colorectal carcinoma; EMT: Epithelial-mesenchymal transition; BCA: Bicinchonininc acid; HUVECs: Human umbilical vein endothelial cells.

## Competing interests

The authors declare that they have no competing interests.

## Authors’ contributions

AGL, ZGZ, MHZ and CXH conceived the study design, participated in its design and in the acquisition of data. JKZ and PL carried out the experiments, participated in the acquisition of data, analysis and interpretation, drafted the manuscript. HF, XZPW, YPZ and JJM has been involved in analyzing the data and drafting the manuscript. ZZ helped to draft and revise the manuscript. All authors read and approved the final manuscript.

## Supplementary Material

Additional file 1: Figure S1 Kaplan-Meier survival analysis of CDH12 expression in 50 CRC patients. The Kaplan-Meier survival analysis showed the survival rate of the CDH12 positive patients group was significantly lower than in the CDH12 negative patients group. Click here for file

Additional file 2: Figure S2 Expression of CDH12 in CRC cell lines and the result of shRNAs sequence screening. A: The Interfering effects of three shRNAs targeting CDH12 in SW1116 and SW620, B: CDH12 expression in CRC cell lines detected by western blot; C: Relative average grey level of western blot bands. Click here for file

Additional file 3: Figure S3 Effect of enforcing CDH12 on cell proliferation and wound healing in CRC cell lines. A: Growth curves of HT29 cell lines were measured by CCK-8 assays. B: Representative photographs of scratch wounds in HT29 (100×), C: Relative distances of cell wounds in CHD12 ectopic expression group and control group (*P<0.05, **P<0.01), D: Growth curves of HT29 cell lines were measured by CCK-8 assays. E: Representative photographs of scratch wounds in HCT116(100×); F: Relative distances of cell CHD12 ectopic expression group and control group (*P<0.05, **P<0.01). Click here for file

Additional file 4: Figure S4 Ectopic expression of CDH12 promotes migration and invasion of CRC cells. A: Representative photographs of migratory or invasive HT29 cells on the membrane. B: Average number of migratory or invasive HT29 cells (*P<0.05). C: Representative photographs of migratory or invasive HCT116 cells on the membrane. D: Average number of migratory or invasive HCT116 cells (*P<0.05). The data represent the mean ± s.d. of three independent experiments. Click here for file
