# Supplementary figures and images for: Cadherin-12 contributes to tumorigenicity in colorectal cancer by promoting migration, invasion, adhersion and angiogenesis
Source: J Transl Med. 2013 Nov 15;11:288. doi: 10.1186/1479-5876-11-288 (PMC3879717; doi:10.1186/1479-5876-11-288)

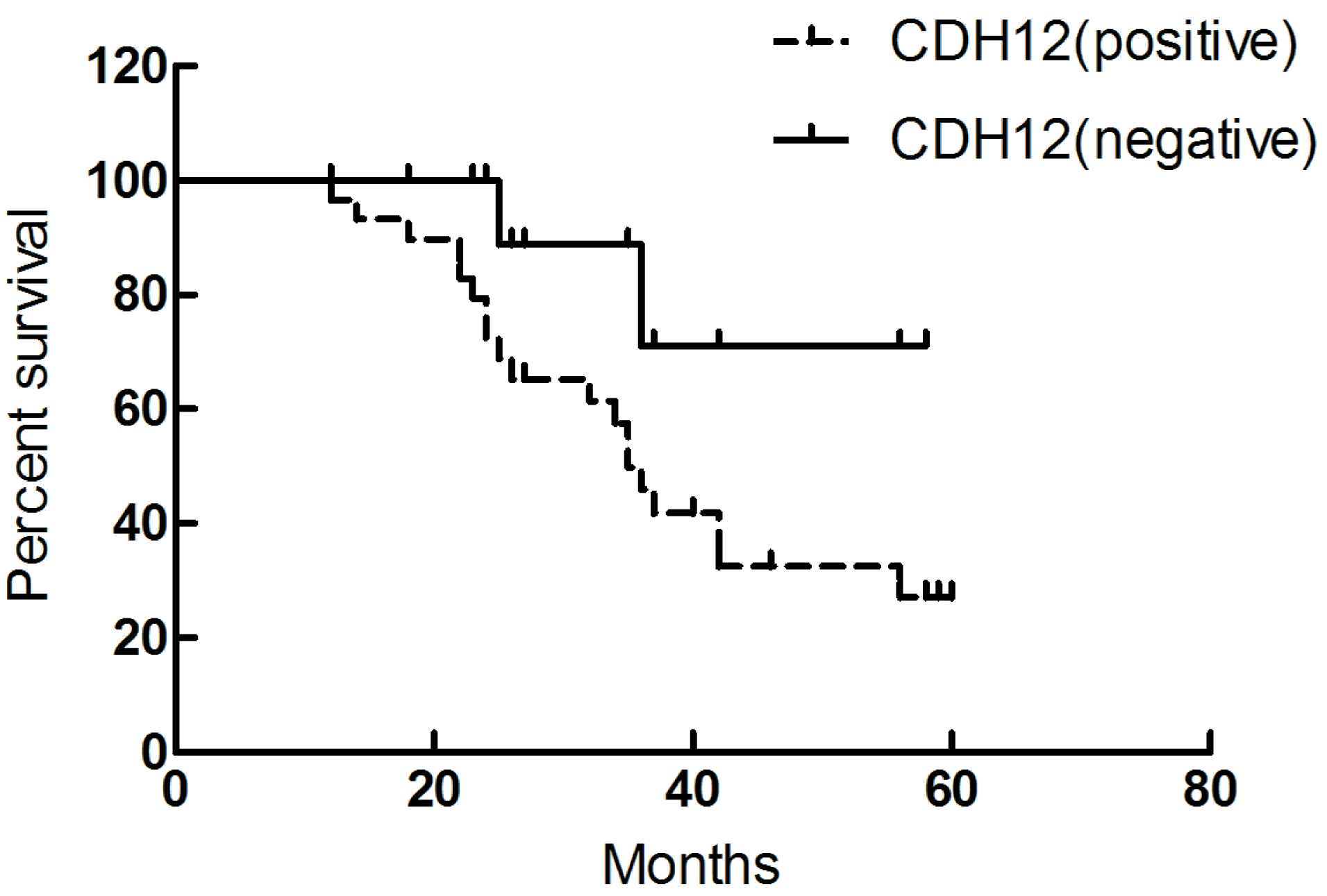

Supplement: Additional file 1: Figure S1 — Kaplan-Meier survival analysis of CDH12 expression in 50 CRC patients. The Kaplan-Meier survival analysis showed the survival rate of the CDH12 positive patients group was significantly lower than in the CDH12 negative patients group. [file 1479-5876-11-288-S1.tiff]

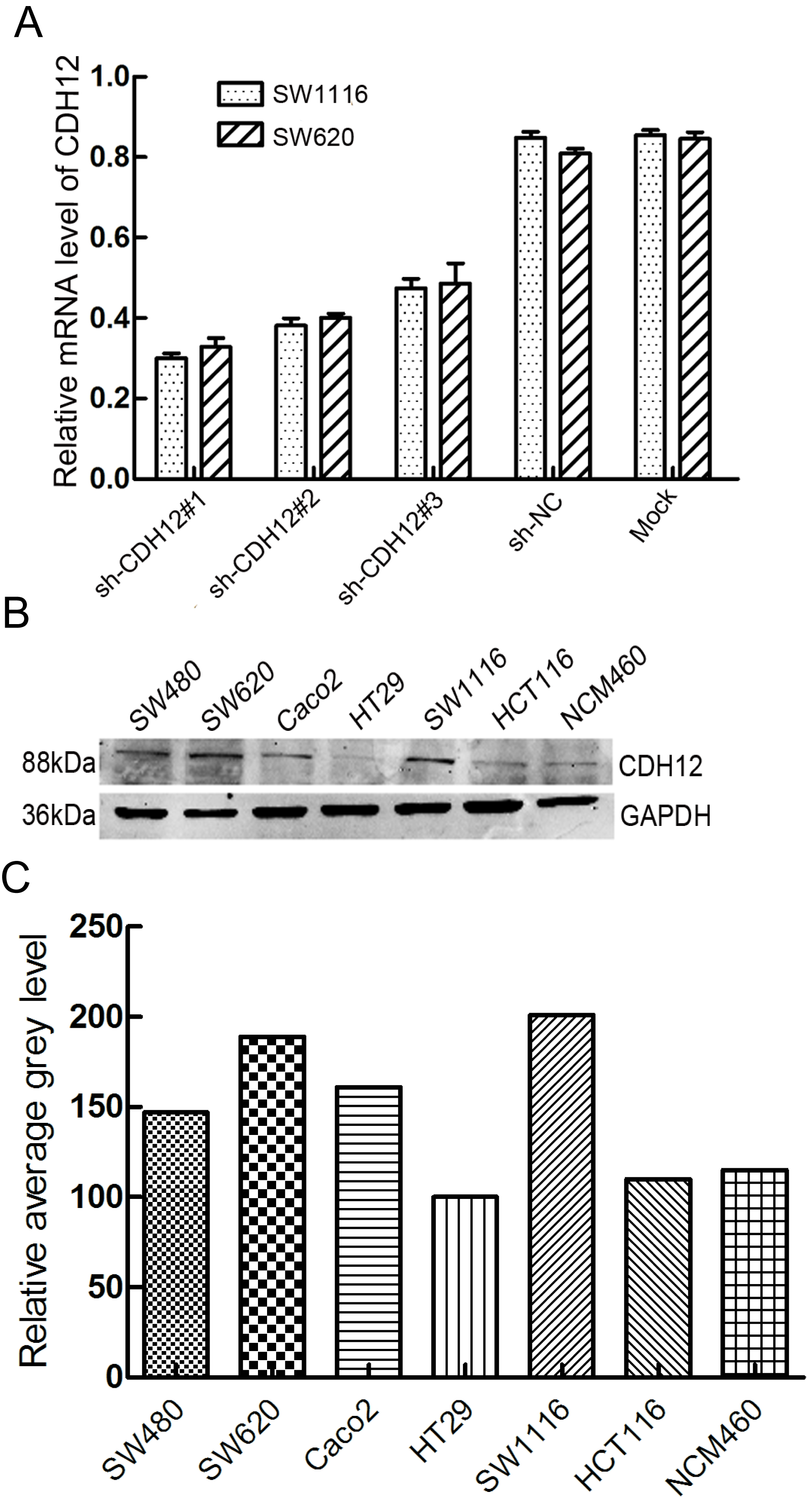

Supplement: Additional file 2: Figure S2 — Expression of CDH12 in CRC cell lines and the result of shRNAs sequence screening. A: The Interfering effects of three shRNAs targeting CDH12 in SW1116 and SW620, B: CDH12 expression in CRC cell lines detected by western blot; C: Relative average grey level of western blot bands. [file 1479-5876-11-288-S2.tiff]

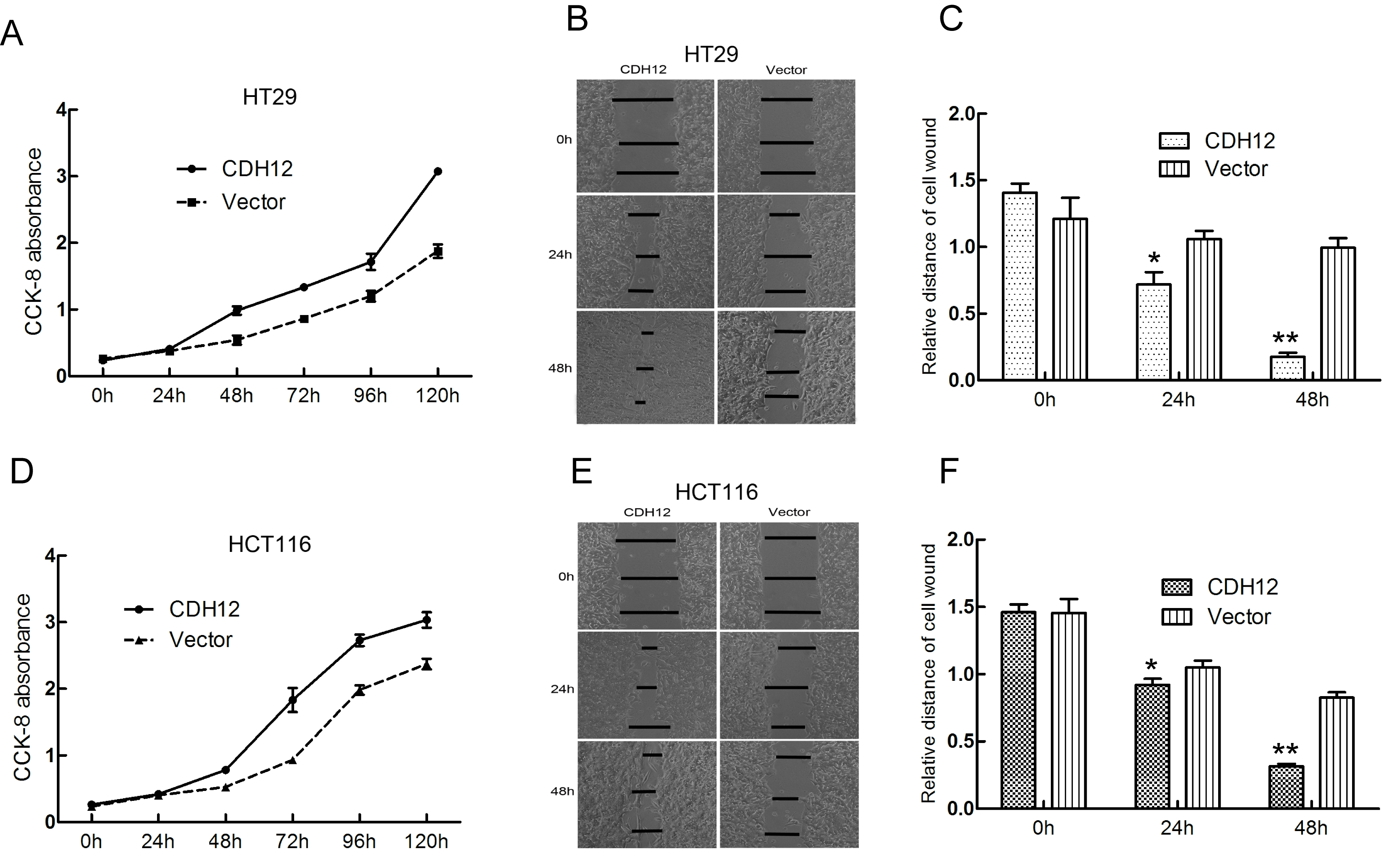

Supplement: Additional file 3: Figure S3 — Effect of enforcing CDH12 on cell proliferation and wound healing in CRC cell lines. A: Growth curves of HT29 cell lines were measured by CCK-8 assays. B: Representative photographs of scratch wounds in HT29 (100×), C: Relative distances of cell wounds in CHD12 ectopic expression group and control group (*P<0.05, **P<0.01), D: Growth curves of HT29 cell lines were measured by CCK-8 assays. E: Representative photographs of scratch wounds in HCT116(100×); F: Relative distances of cell CHD12 ectopic expression group and control group (*P<0.05, **P<0.01). [file 1479-5876-11-288-S3.tiff]

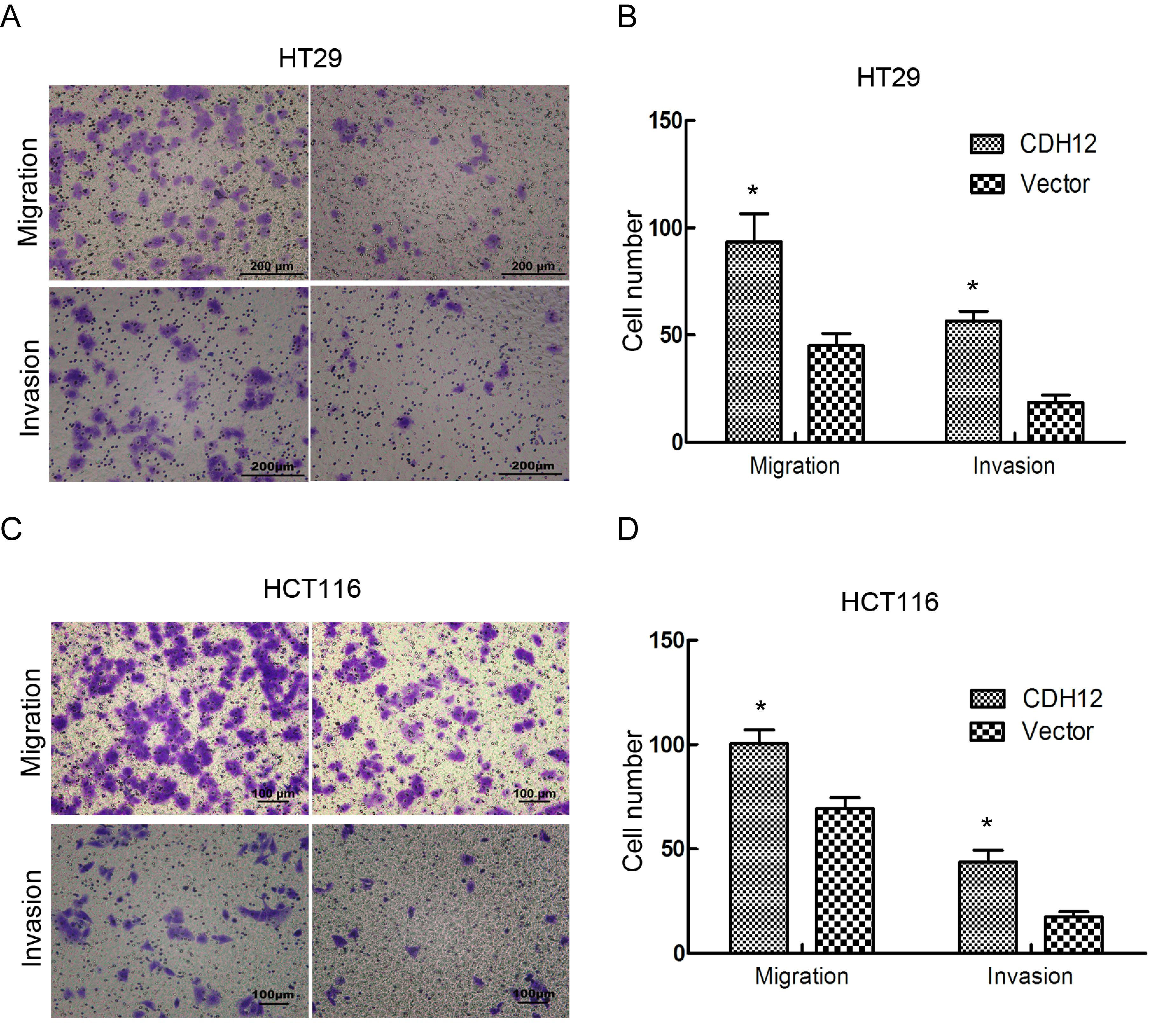

Supplement: Additional file 4: Figure S4 — Ectopic expression of CDH12 promotes migration and invasion of CRC cells. A: Representative photographs of migratory or invasive HT29 cells on the membrane. B: Average number of migratory or invasive HT29 cells (*P<0.05). C: Representative photographs of migratory or invasive HCT116 cells on the membrane. D: Average number of migratory or invasive HCT116 cells (*P<0.05). The data represent the mean ± s.d. of three independent experiments. [file 1479-5876-11-288-S4.tiff]
